# Species, antibiotic susceptibility profiles and *van* gene frequencies among enterococci isolated from patients at Mulago National Referral Hospital in Kampala, Uganda

**DOI:** 10.1186/s12879-019-4136-7

**Published:** 2019-05-31

**Authors:** David P. Kateete, Moses Edolu, Edgar Kigozi, Jeffrey Kisukye, Hannington Baluku, Frank Nobert Mwiine, Christine F. Najjuka

**Affiliations:** 1 Department of Immunology and Molecular Biology, Makerere University College of Health Sciences, Kampala, Uganda; 2 Department of Biomolecular Resources & Biolab Sciences, School of Biosecurity, Biotechnical & Laboratory Sciences, College of Veterinary Medicine, Animal Resources and Biosecurity, Makerere University, Kampala, Uganda; 3 Department of Medical Microbiology, Makerere University College of Health Sciences, Kampala, Uganda

**Keywords:** Enterococcus, *Enterococcus faecium*, *Enterococcus faecalis*, Vancomyicin resistant enterococci, VRE, *vanA*, *vanB*, Mulago hospital, Kampala-Uganda

## Abstract

**Background:**

The increase in drug resistance to affordable antibiotics used to treat Gram positive bacterial infections has complicated the management of enterococcal infections. Resistance to vancomycin, one of the most powerful antibiotics, is of utmost concern as both intrinsic and acquired forms of resistance do occur in enterococci. This cross-sectional study aimed to determine the species, antibiotic susceptibility profiles and *vanA*/*vanB* gene frequencies among enterococci isolated from patients at Mulago Hospital in Kampala, Uganda.

**Methods:**

Between November 2011 and October 2012, stool, urine, sputum and blood samples, as well as vaginal, endocervical, pus, ear and urethra swabs from 3229 patients were processed for isolation of bacteria, yielding 162 enterococci of which 115 were available for analysis (one isolate per specimen/patient). Species-level confirmation and susceptibility testing were determined with the Phoenix™ AST/ID Automated System, while *vanA*/*vanB* gene carriage was determined by PCR.

**Results:**

Species-level identification revealed 72 isolates of *E. faecalis*, 20 *E. gallinarum/casseliflavus,* 5 *E. faecium,* 4 *E. raffinosus* and 2 isolates each for *E. hirae* and *E. durans*. Ten isolates could not be identified to species level. Antibiotic resistance was generally low especially to ampicillin, quinolones, nitrofurantoin, glycopeptides and linezolid, but high for erythromycin and tetracycline. Equally, *vanA* and *vanB* gene frequencies were low (i.e. 15.8 and 7.9%, respectively) and detected only in *E. casseliflavus/gallinarum* species that are intrinsically resistant to vancomycin*.* Vancomycin resistant isolates of *E. faecalis* and *E. faecium* were not detected.

**Conclusions:**

Enterococcus species are frequent in clinical specimens at Mulago Hospital but they are highly susceptible to common antibiotics especially ampicillin. While vancomycin resistant enterococcal (VRE) isolates of *E. faecium* and *E. faecalis* are rare in the hospital and frequency of multidrug resistance is low, non-*faecium* and non-*faecalis* VRE isolates (i.e. *E. gallinarum*/*casseliflavus*) are frequent, some with VanA/VanB (high-level) vancomycin resistance. Therefore, species-level identification of enterococci is necessary in resource limited settings to guide infection control and treatment of enterococcal infections.

**Electronic supplementary material:**

The online version of this article (10.1186/s12879-019-4136-7) contains supplementary material, which is available to authorized users.

## Background

The enterococci are a diverse and versatile group of Gram positive, lactic acid producing bacteria, of which *Enterococcus faecalis* and *E. faecium* are the most predominant species. While they primarily inhabit the gastrointestinal tract of humans and other mammals, enterococci are also widely distributed in nature: they can be found in soil, water, dairy products and other foodstuffs, and on plants [1, 2]. They are able to thrive under a variety of conditions and this creates a constant source of infection. Because of their inherent and acquired antibiotic resistance mechanisms, and ability to survive in harsh environments in community and hospital settings [3], enterococci have emerged as major causes of difficult-to-treat nosocomial and community acquired infections particularly biliary and urinary tract infections, bacteremia, endocarditis, intra-abdominal infections, pelvic infections, etc.

As mentioned, enterococci are intrinsically resistant to a variety of antibiotics especially cephalosporins, sulfonamides, oxacillin, ertapenem and perfloxacin. Specifically, *E. faecalis* and *E. faecium* are intrinsically resistant to cephalosporins, aminoglycosides, clindamycin, trimethoprim-sulfamethoxazole and fusidic acid; yet again *E. faecalis* is intrinsically resistant to quinupritin. Moreover, through mutation and horizontal gene transfer (HGT) processes, enterococci are able to acquire additional resistance mechanisms to key antibiotics notably tetracycline, erythromycin, fluoroquinolones, rifampicin, chloramphenicol, nitrofurantoin, fusidic acid, glycopeptides (vancomycin & teicoplanin) and to high concentrations of aminoglycosides and β-lactams [4]. While *E. gallinarum* and *E. casseliflavus* are less pathogenic than *E. faecalis* and *E. faecium* and they are highly susceptible to ampicillin, they are intrinsically resistant to one of the most potent antibiotics, vancomycin [4–6]. Furthermore, the hospitalization of patients and progress in medical technology and treatment, as well as increase in antibiotic usage, have contributed to a surge in infections due to multidrug resistant (MDR) enterococci and vancomycin resistant enterococci (VRE). VRE are of utmost concern in that vancomycin is a powerful antibiotic used to treat Gram positive bacterial infections yet, both intrinsic and acquired forms of resistance do occur in enterococci. VRE have become an important cause of serious invasive infections globally to such an extent that clinical microbiology laboratories are encouraged to speciate enterococcal isolates from hospitals and screen them for vancomycin resistance [4, 6].

Glycopeptide resistance in enterococci is of two main phenotypes [4, 6]. The first and most common vancomycin resistance phenotype among VRE is high-level resistance that occurs through acquisition of *vanA* & v*anB* genes, usually in *E. faecium* and *E. faecalis*, the species comprising majority of enterococcal infections especially bloodstream/invasive VRE infections. Intrinsic resistance to vancomycin also occurs in enterococci, characteristically in *E. gallinarum* and *E. casseliflavus.* These species possess the VanC enzymes, which synthesize pentapeptide peptidoglycan precursors ending in D-alanyl-D-serine that have reduced affinity for vancomycin hence, they are the source of intrinsic vancomycin resistance in *E. gallinarum*/*casseliflavus* [4, 6]. Note, high-level vancomycin resistance of the VanA/VanB phenotype is transferrable and it occurs in *E. gallinarum*/*casseliflavus* [4–6]. Overall, VRE other than *E. faecium* and *E. faecalis* are infrequently associated with infections but their frequencies are likely to be underreported in the developing countries.

Henceforth, it has become necessary to screen enterococci isolated from hospitalized patients for vancomycin resistance, especially isolates from patients with endocarditis and meningitis, and isolates from sterile body parts [4]. VRE speciation is particularly important as it allows differentiation of *vanC* carrying organisms and avoid misidentifying them as VanA/VanB VRE. Furthermore, as colonization of the gut and other body parts by the enterococci is a key predisposing factor for infection and it is the main source of endogenous infection by enterococci [2], characterizing enterococci from clinical specimens is proxy for understanding enterococcal colonization, and it generates insight into their infection dynamics in specific settings.

Although enterococci are amongst the leading causes of urinary tract infections (UTIs) and bacteremia worldwide [7], their significance in Uganda is not clear as there are no clearly defined rates for enterococcal infections in this country. All the same, preliminary estimates for enterococcal infections reveal high rates for UTIs and surgical site infections (SSIs) in Uganda [8, 9]. The aim of this study was to define the species and antibiotic susceptibility profiles of enterococci isolated from patients at Mulago National Referral Hospital in Kampala, Uganda. We also estimated the *vanA* and *vanB* gene frequencies among the isolates.

## Methods

### Study setting, specimens and isolates

This was a cross-sectional study conducted at the Clinical Microbiology Laboratory of the Department of Medical Microbiology, Makerere University College of Health Sciences. The molecular assays were performed at the Molecular Diagnostics Laboratory of the Department of Immunology & Molecular Biology, Makerere University College of Health Sciences. Between December 2011 and November 2012, the Clinical Microbiology Laboratory processed 3229 clinical specimens from approx. The same number of patients (i.e. one specimen per patient) at Mulago National Referral Hospital, for isolation of bacteria. The specimens processed included blood 34% (1094/3229), sputum 24% (777/3229), high vaginal swabs 9% (287/3229), urine 7.8% (252/3229), endo-cervical swabs 7.8% (252/3229), stool 7.4% (238/3229), pus swabs 4.1% (133/3229), ear swabs 1.3% (42/3229), urethra swabs 1.3% (42/3229), cerebrospinal fluid 1.3% (42/3229), lymph node aspirates 0.65% (21/3229), oral swabs 0.43% (14/3229), rectal swabs 0.22% (7/3229), tracheal aspirates 0.22% (7/3229), broncho-alveolar lavage 0.22% (7/3229), pharyngeal swabs 0.22% (7/3229) and pleural fluids 0.22% (7/3229), Additional file 1: Table S1. These specimens were processed on request by clinicians during routine clinical investigations.

Of the 3229 specimens, Gram positive and catalase negative isolates grew from a total of 196 specimens (one isolate per specimen). All the 196 Gram positive and catalase negative bacterial isolates had a diplococcal chain appearance under microscopy, and they were subjected to growth on bile esculin agar (BEA), a selective differential medium used to identify members of the genus *Enterococcus*. Additionally, a 6.5% (w/v) sodium chloride tolerance test was carried out in order to differentiate enterococci from group D streptococci. Following these two procedures, a total of 162 pure isolates (one per specimen) were presumptively confirmed to be enterococci and stored at − 80 °C.

Presumptive species-level identification was based on motility and pigmentation, and formation of acid in mannitol, sorbitol, sucrose, arabinose and pyruvate broths [6]. Briefly for the pyruvic acid test, pyruvate broth was inoculated with a single colony from a 24 h pure culture, incubated aerobically at 35 °C with a loose cap and examined daily for 3–5 days. Acid production was confirmed by color change in the bromothymol indicator from blue-green to yellow, which was interpreted as positive for *E. faecalis*. *E. faecalis* ATCC51299 was used as the positive control for the pyruvate test. For the arabinose test, Brain Heart Infusion (BHI) broth containing arabinose and bromocresol purple indicator was inoculated with a single colony from a 24 h pure culture, and incubated overnight at 35 °C. Color change in the media to yellow was interpreted as positive for *E. faecium* [10]. For motility test, microscopy was done to presumptively differentiate *E. gallinarum* from other enterococci.

### Species confirmation and antimicrobial susceptibility testing

**Fig. 1:**
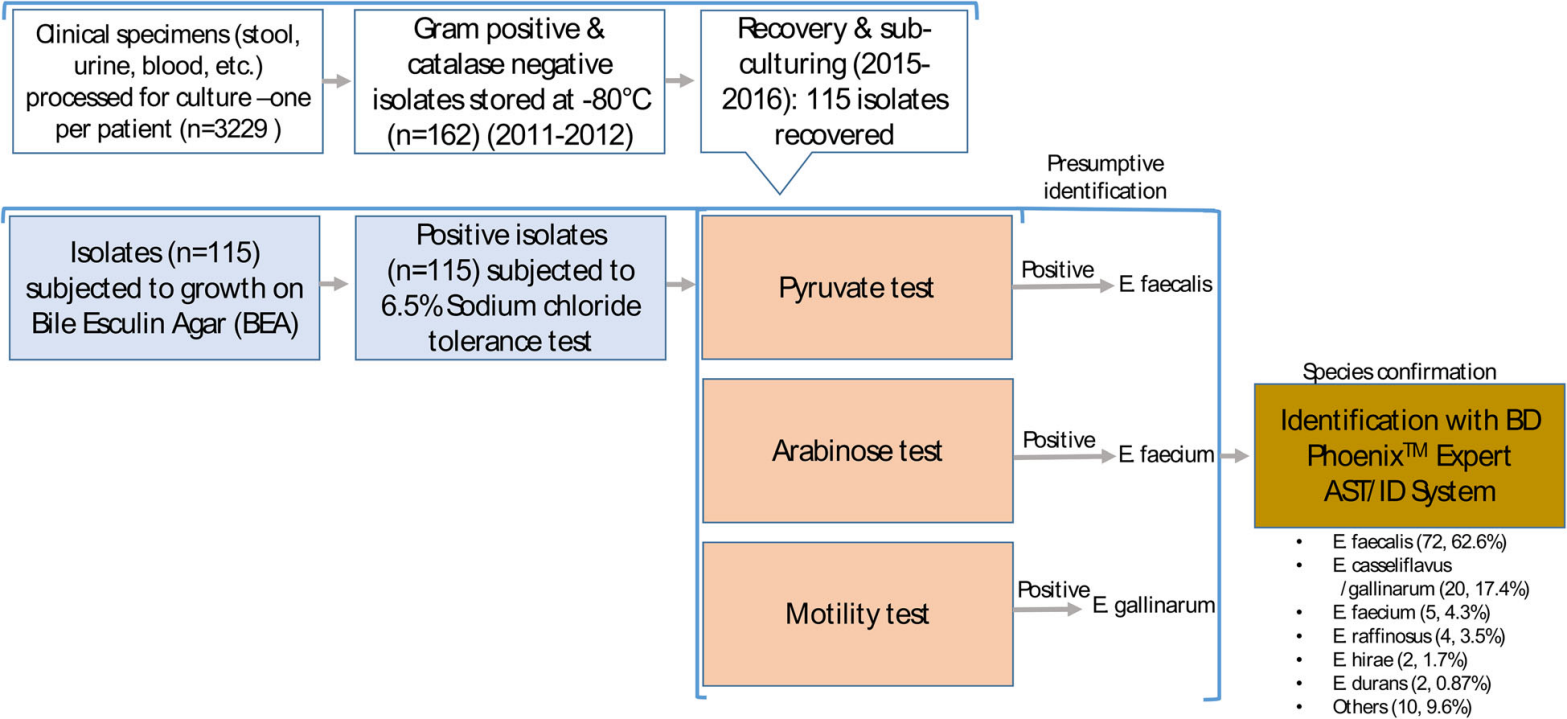
Study flow chart illustrating our strategy for identification of Enterococcus both at genus and species levels

Species-level confirmation and antimicrobial susceptibility testing (AST) by minimum inhibitory concentration (MIC) were achieved by the BD Phoenix™ Automated Identification & Susceptibility Testing system according to the manufacturer’s recommendations. The BD Phoenix™ default MIC breakpoints to a panel of 11 antibiotics to which enterococci are susceptible were used: ampicillin ≤2 μg/ml, daptomycin 4 μg/ml, teicoplanin ≤1 μg/ml, vancomycin ≤0.5 μg/ml, erythromycin ≤0.25 μg/ml, linezolid ≤1 μg/ml, nitrofurantoin 64 μg/ml, ciprofloxacin ≤0.5 μg/ml, moxifloxacin ≤0.5 μg/ml, tetracycline ≤0.5 μg/ml, and gentamicin-syn ≤500 μg/ml. For quality control, *E. faecalis* ATCC29212 and *E. faecium* HA56038 (ATCC) were included in the Phoenix AST & ID panels. Figure 1 depicts the diagnostic strategy we used to identify enterococci to genus and species levels.

### Genotyping

To detect the *vanA* and *vanB* genes in enterococci, PCR was performed on a Techne TC-412 thermocyler in reaction volumes of 12.5 μl using previously published primers [11] 5′-AATGTGCGAAAAACCTTGCG-3′ & 5′-CCGTTTCCTGTATCCGTCC-3′ (for *vanA*); 5′-CAAATCACTGGCCTACATTC-3′ & 5′-TCTGCATCCAAGCACCCG-3′ (for *vanB*). Each reaction contained 5 μl of 2xTaq master mix (Sigma Co.), 1 μl of forward and reverse primers, 3 μl nuclease free water and 2.5 μl of crude enterococcal DNA extract (template). The cycling conditions were as follows: Initial denaturation at 94 °C, 4 min followed by 31 cycles of 94 °C 1 min, 50 °C 1 min, and 68 °C 1 min, and a final extension step at 68 °C, 10 min. The PCR products were analyzed by agarose gel electrophoresis with ethidium bromide staining (0.5 μg/ml). Repetitive elements-based PCR (Rep-PCR) genotyping for insight into genetic relatedness of the isolates was performed as previously described [12].

## Results

### Frequency of Enterococcus species

We processed a total of 3229 specimens from 3229 patients, yielding 162 Gram positive and catalase negative isolates (one isolate per patient/specimen), of which 115 were available for analysis. Females were 2066 (64%) and the median age of the patients with samples yielding enterococci was 28 years (Interquartile Range (IQR) of 19 to 44). All the 115 isolates grew on BEA and survived in BHI broth supplemented with 6.5% sodium chloride hence, they were presumptively identified as enterococci, Fig. 1*.* Therefore, the overall frequency of enterococci in the processed clinical samples was 17.6% (42/238) stool, 10% (25/252) urine, 1.9% (21/1094) blood, 1.93% (15/777) sputum, 7.5% (10/133) pus swabs and 4.8% (02/42) ear swabs. The 47 (29%, 47/162) stored isolates that were not recovered on sub-culturing were mainly from vaginal swabs (27), urine (7), stool (3), endo-cervical swabs (2), urethra swabs (7) and rectal swabs (1), Additional file 1: Table S1. Once more, there was no enterococcus growth from cerebrospinal fluids, lymph node aspirates, tracheal aspirates, bronchoalveolar lavage, pleural fluids, oral and pharyngeal swabs, Additional file 1: Table S1. Species-level identification of the enterococcus with the BD Phoenix™ AST/ID system yielded 72 isolates of *E. faecalis*, 20 *E. gallinarum/casseliflavus,* 5 *E. faecium,* 4 *E. raffinosus,* and 2 isolates each for *E. hirae* and *E. durans*. Ten isolates could not be identified to species level, Table 1.

**Table 1:** Enterococcus isolates identified to species level

Species	Number of isolates^a^	Percent
*Enterococcus faecalis*	72	62.6
*Enterococcus casseliflavus / gallinarum*	20	17.4
*Enterococcus faecium*	05	4.3
*Enterococcus raffinosus*	04	3.5
*Enterococcus hirae*	02	1.7
*Enterococcus durans*	02	0.87
Others	10	9.6
Total	115	100

^a^One isolate per specimen

### Antibiotic susceptibility profiles and *van* gene frequencies

Antibiotic resistance was generally low for all the species especially to ampicillin, quinolones (ciprofloxacin & moxifloxacin), gentamicin-syn, nitrofurantoin, glycopeptides, linezolid and daptomycin but high for erythromycin and tetracycline, Table 2. All *E. faecalis* isolates were susceptible to nitrofurantoin, vancomycin, teicoplanin and linezolid but resistant to erythromycin and tetracycline, Table 2. Further, all but one isolate of *E. faecalis* that were intermediate or resistant to ciprofloxacin were also resistant to tetracycline, and all except two were resistant to erythromycin. Of the three isolates that were resistant to gentamicin-syn, two were *E. faecalis* (Table 2) one of which exhibited multiple resistance to several antibiotics (i.e. ampicillin, erythromycin, nitrofurantoin, ciprofloxacin, tetracycline) but susceptible to teicoplanin, vancomycin and daptomycin. For *E. faecium*, all the five isolates were susceptible to gentamicin-syn, ampicillin, vancomycin, teicoplanin, linezolid and moxifloxacin however, four were non-susceptible to daptomycin. One isolate of *E. faecium* was resistant to both erythromycin and nitrofurantoin and intermediate to ciprofloxacin.

**Table 2:** Antimicrobial susceptibility profiles of enterococcal isolates investigated

Drug	*E. faecalis*, *n* = 72			*E. faecium*, *n* = 05			Others, *n* = 38		
	R (%)	I (%)	S (%)	R (%)	I (%)	S (%)	R (%)	I (%)	S (%)
Erythromycin	54 (72)	07 (9.7)	11 (15.3)	04 (80)	0	01 (20)	07 (18.4)	04 (10.5)	11 (28.9)
Tetracycline	50 (69.4)	0	22 (30.6)	03 (60)	0	02 (40)	05 (13.2)	0	33 (86.8)
Ciprofloxacin	07 (9.7)	06 (8.3)	59 (81.9)	03 (60)	01 (20)	01 (20)	x	x	x
Moxifloxacin	01 (0.14)	0	71 (98.6)	0	0	05 (100)	04 (10.5)	04 (10.5)	30 (78.9)
Ampicillin	01 (1.4)	0	71 (98.6)	0	0	05 (100)	02 (5.3)	0	36 (94.7)
Gentamicin-Syn	02 (2.8)	0	72 (100)	0	0	05 (100)	01 (2.6)	0	01 (2.6)
Nitrofurantoin	0	0	72 (100)	01 (20)	01 (20)	03 (60)	01 (2.6)	03 (7.9)	34 (89.5)
Vancomycin	0	0	72 (100)	0	0	05 (100)	20 (52.6)	0	18 (47.4)
Teicoplanin	0	0	72 (100)	0	0	05 (100)	03 (7.9)	0	35 (92.1)
Linezolid	0	0	72 (100)	0	0	05 (100)	01 (2.6)	04 (10.5)	34 (89.5)
Daptomycin	0	0	64 (88.9)	0	0	01 (20)	0	0	07 (18.4)
MDR		NA	NA		NA	NA		NA	NA
*vanA*	–	–	–	–	–	–	06 (15.8)	–	–
*vanB*	–	–	–	–	–	–	03 (7.9)	–	–

*S* Susceptible, *I* Intermediate, *R* Resistant; −,Negative; x –MICs for these species (see “others”) are not reported by the Phoenix system

MDR, multidrug resistant (isolate resistant to three or more antibiotic classes)

“Others” denotes *E. casseliflavus/gallinarum*, *E. raffinosus*, *E. hirae*, *E. durans*, and the 10 isolates that were not identified to species level

Furthermore, all the other species combined (i.e. *E. casseliflavus/gallinarum, E. raffinosus, E. hirae, E. durans* and the 10 isolates that were not identified to species level) exhibited varied susceptibilities to antimicrobials with a total of 31 isolates being non-susceptible to daptomycin. Overall, vancomycin resistance in this study was detected only in the 20 isolates of *E. casseliflavus/gallinarum* (Table 2) that are intrinsically resistant to this drug. Likewise, resistance to teicoplanin was detected in only *E. casseliflavus/gallinarum* at 15% (3/20); however, the teicoplanin resistant isolates were susceptible to ampicillin, erythromycin, nitrofurantoin, tetracycline and gentamicin-syn. Once more, the only linezolid resistant isolate was *E. casseliflavus/gallinarum*; it was also resistant to vancomycin, teicoplanin and moxifloxacin but susceptible to ampicillin, tetracycline, nitrofurantoin and gentamicin-syn.

### *van* gene frequencies and multidrug resistance phenotype

Generally the *vanA* and *vanB* gene frequencies were low i.e. 15.8 and 7.9% respectively, and detected in only *E. casseliflavus/gallinarum* species, Table 3 & Additional file 2: Figure S1*.* The species and resistance patterns of enterococci resistant to two or more antibiotics are summarized in Table 4. Regarding resistance to antibiotics to which enterococci are commonly susceptible (i.e. erythromycin, tetracycline, ciprofloxacin, moxifloxacin, ampicillin, nitrofurantoin, vancomycin, teicoplanin, linezolid, daptomycin), only 11 isolates (9.6%, 11/115) were MDR and almost all the MDR patterns involved resistance to tetracycline, Table 4. Majority of MDR isolates belonged to *E. faecalis* (five isolates) and *E. casseliflavus/gallinarum* (four isolates) while the remaining two isolates were *E. raffinosus* and *E. durans*. Besides MDR, the most common resistance pattern among isolates resistant to two or more antibiotics involved resistance to erythromycin and tetracycline, Table 4. Lastly, cluster analysis of the Rep-PCR fingerprints revealed no evidence of clonal spread/transmission of enterococci in the Mulago Hospital setting (not shown).

**Table 3:** Antibiotic susceptibility profiles of vancomycin resistant *E. casseliflavus/gallinarum*

Isolate #	AMP	GEN	ERY	TET	MXF	NIT	DAP^a^	LIZ	TEC	VAN	*vanA*	*vanB*
EM0085	S	S	R	R	S	S	N	S	S	R	–	–
EM0176	S	S	R	S	S	I	N	S	S	R	+	–
EM3113	S	S	R	R	S	S	N	S	S	R	–	–
EM0476	S	S	S	S	I	S	N	S	R	R	+	–
EM2016	S	S	R	R	S	S	S	S	S	R	–	+
EM0040	S	S	S	S	R	S	N	S	S	R	–	–
EM0049	S	S	S	S	R	S	N	S	S	R	+	–
EM0149	S	S	S	S	I	S	S	S	S	R	–	–
EM0477	S	S	S	S	I	S	S	I	R	R	+	–
EM1161	S	S	S	S	S	S	S	S	S	R	–	+
EM0162	S	S	I	S	R	S	N	R	R	R	+	–
EM0074	S	S	S	S	I	S	S	S	S	R	–	–
EM0014	S	S	S	S	S	S	S	S	S	R	–	–
EM0116	S	S	S	S	S	S	S	S	S	R	–	+
EM0022	S	S	S	S	S	S	S	S	S	R	–	–
EM0056	S	S	S	S	S	S	S	S	S	R	–	–
EM0099	S	S	S	S	S	S	N	S	S	R	–	–
EM0010	S	S	S	S	S	S	S	S	S	R	+	–
EM0018	S	S	S	S	S	S	S	S	S	R	–	–
EM1009	S	S	S	S	S	S	S	S	S	R	–	–
Total R/+ (%)	0	0	04 (20)	03 (15)	03 (15)	0	0	01 (5)	03 (15)	20 (100)	06 (30)	03 (15)

*E. casseliflavus* & *Enterococcus gallinarum* are intrinsically resistant to vancomycin

*AMP* ampicillin, *VAN* vancomycin; ^a^*GEN* high level gentamicin, *ERY* erythromycin, *TET* tetracyclin, *MXF* moxifloxacin, *NIT* nitrofurantoin, *DAP* daptomycin, *TEC* teicoplanin, *LIZ* linezolid, *S* Susceptible, *I* Intermediate, *R* Resistant, *N* non-susceptible; −, Negative; +, Positive

**Table 4:** Species and resistance patterns of enterococci resistant to two or more antibiotics

Isolate ID	Species	Resistance pattern	MDR phenotype
EM3132	*E. faecalis*	ERY-GEN-AMP-CIP-MXF-TET	Yes
EM2591	*E. faecalis*	ERY-GEN-TET	Yes
EM0082	*E. faecalis*	ERY-CIP-TET	Yes
EM0028	*E. faecalis*	ERY-CIP-TET	Yes
EM1239	*E. faecalis*	ERY-CIP-TET	Yes
EM0057	*E. faecalis*	ERY-TET	No
EM0055	*E. faecalis*	ERY-TET	No
EM0270	*E. faecalis*	ERY-TET	No
EM0098	*E. faecalis*	ERY-TET	No
EM0056	*E. faecalis*	ERY-TET	No
EM0066	*E. faecalis*	ERY-TET	No
EM0073	*E. faecalis*	ERY-TET	No
EM1196	*E. faecalis*	ERY-TET	No
EM2591	*E. faecalis*	ERY-TET	No
EM0216	*E. faecalis*	ERY-TET	No
EM0141	*E. faecalis*	ERY-TET	No
EM0011	*E. faecalis*	ERY-TET	No
EM2970	*E. faecalis*	ERY-TET	No
EM1023	*E. faecalis*	ERY-TET	No
EM0033	*E. faecalis*	ERY-TET	No
EM0142	*E. faecalis*	ERY-TET	No
EM0333	*E. faecalis*	ERY-TET	No
EM1333	*E. faecalis*	ERY-TET	No
EM0297	*E. faecalis*	ERY-TET	No
EM0016	*E. faecalis*	CIP-TET	No
EM0087	*E. faecalis*	CIP-TET	No
EM0175	*E. faecium*	ERY-CIP	No
EM0154	*E. raffinosus*	GEN-ERY-TET	Yes
EM0704	*E. durans*	ERY-AMP-NIT-MXF-TET	Yes
EM0085	*E. gallinarum/casseliflavus*	VAN*-ERY-TET	Yes
EM3113	*E. gallinarum/casseliflavus*	VAN*-ERY-TET	Yes
EM2016	*E. gallinarum/casseliflavus*	VAN*-ERY-TET	Yes
EM0162	*E. gallinarum/casseliflavus*	VAN*-TEC-LIZ-MXF	Yes
EM0476	*E. gallinarum/casseliflavus*	VAN*-TEC	No
EM0477	*E. gallinarum/casseliflavus*	VAN*-TEC	No
EM0176	*E. gallinarum/casseliflavus*	VAN*-ERY	No
EM0040	*E. gallinarum/casseliflavus*	VAN*-MXF	No
EM0049	*E. gallinarum/casseliflavus*	VAN*-MXF	No
Total MDR (%)			11 (9.6)

*AMP* ampicillin, *VAN* vancomycin, *GEN high level gentamicin, *ERY* erythromycin, *TET* tetracyclin, *MXF* moxifloxacin, *CIP* Ciprofloxacin, *NIT* nitrofurantoin, *DAP* daptomycin, *TEC* teicoplanin, *LIZ* linezolid

*E. gallinarum/casseliflavus are intrinsically resistant to vancomycin

## Discussion

In this study, one of the most predominant *Enterococcus* species, *E. faecalis* [4, 13, 14], was the most frequent at 62.6%. However *E. faecium*, another key enterococcus species worldwide especially in hospital settings, was not frequent. The reported low frequency for *E. faecium* and several other enterococcus species in this study (*E. durans*, *E. hirae* and *E. raffinosus*) is in line with global reports for these species [4] in that up to 80–90% of the enterococcal infections in humans are attributed to *E. faecalis*, 10–15% *E. faecium* and < 5% combined other species [6]. Note, the proportion of *E. faecium* in this study was twofold lower than expected. While this could have resulted from a potential sampling bias, *E. faecium* being significantly less frequent than *E. faecalis* at Mulago Hospital has previously been reported i.e. prevalence of 8.7% (2/23) and 91.3% (21/23) for *E. faecium* and *E. faecalis*, respectively [9].

Interestingly, the second most frequent enterococcus species in this study were the *E. gallinarum*/*casseliflavus* species at 17.4%, which is higher than expected from global reports for these species (i.e. ≤2%) [5, 6]. Although *E. gallinarum*/*casseliflavus* are infrequently isolated from clinical specimens, especially in the developed countries, several reports have implicated them as causes of serious invasive infections [5, 6]. These species colonize the gut of hospitalized and non-hospitalized people with overall colonization rates ranging from 5.7–12.1% [6]. The current study, and recent findings from Ethiopia [15] and Sri Lanka [16], suggest that the colonization rate for these species is high in developing countries. However, as we investigated isolates from symptomatic individuals, our findings should not be used to draw inferences on enterococcus colonization in the wider population. The clinical and epidemiological features of cases of *E. casseliflavus/gallinarum* bacteremia in the United States were reported [5, 6], with underlying conditions such as immunodeficiency, malignancy, receipt of transplant etc. reported in up to 95% of the patients [5, 6]. Of all the risk factors investigated only the immunocompromised status was a significant predictor of mortality in patients with *E. gallinarum*/*casseliflavus* bacteremia [5]. Perhaps, the rampant poverty in Uganda and immunodeficiency linked to prevalent HIV-infection and malnutrition are factors that could be responsible for high occurrence of *E. gallinarum*/*casseliflavus* in patients at Mulago Hospital, though this requires further investigation. Relatedly, the high frequency of *E. gallinarum*/*casseliflavus* species in this study was comparable to rates from Ethiopia (26.3% *E. gallinarum*/*casseliflavus*, 17.5% *E. gallinarum* and 8.8% *E. casseliflavus*) [15]), a low-income country like Uganda with high HIV-infection rates.

Almost all the enterococci in this study were susceptible to ampicillin (only three isolates were resistant) and this is in agreement with the finding that enterococci are generally susceptible to ampicillin [4]. Note, ampicillin resistance in this study was significantly lower than rates from Ethiopia [15] and India [17], but this could be due to differences in methods for drug susceptibity testing e.g. we used MICs by an automated instrument that are generally more efficient compared to the disk diffusion methods used by investigators in Ethiopia [15]. As ampicillin is the drug of choice in treatment of enterococcal infections, the high susceptibility of isolates implies that this cheap antibiotic is effective in treatment of enterococcal infections in Uganda. Only three isolates exhibited high-level resistance to gentamicin, two of which were *E. faecalis* and these are few compared to rates from Ethiopia [15]. At the moment, high-level gentamicin resistance in enterococci may not be a cause for concern in Uganda however, continuous surveillance in hospital settings is necessary to monitor this trend. Further, several isolates in this study were non-susceptible to daptomycin, and this could be attributed to tolerance (“the ability of the organism to survive levels of drugs well in excess of the MIC” [4]), a common finding among clinical isolates of enterococci [4].

All the *E. faecalis* and *E. faecium* isolates in this study were susceptible to vancomycin and the only VRE isolates detected were *E. gallinarum/casseliflavus* species that are inherently resistant to vancomycin. Elsewhere, investigators in Spain who studied the antibiotic resistance of enterococci from healthy breastfed infants also did not detect VRE isolates of *E. faecalis* and *E. faceicum* [18]. As the VRE isolates (i.e. *E. gallinarum/casseliflavus*) in this study were susceptible to ampicillin, this antibiotic should be the drug of choice for treating enterococcal infections in Uganda as increased use of vancomycin may lead to selection of glycopeptide resistant enterococci in a resource-limited setting. It is important to note that lack of VRE isolates of *E. faecalis*/*faecium* in this study may not imply they are rare in Uganda: a VRE isolate of *E. faecalis* was recovered from bovine milk samples in Kampala [19] and from a patient with a SSI at Mulago Hospital [9].

The *vanA* and *vanB* genes were not detected in *E. faecalis* and *E. faecium*, which was expected as these genes encode high-level vancomycin resistance [20] that was not detected in *E. faecalis*/*faecium* isolates. Yet, *vanA*/*vanB* occurred in *E. gallinarum/casseliflavus*; although vancomycin resistance in these species is reported to be of the low-level type [4–6], their detection implies that acquisition of the *vanA*/*vanB* genes occurred, most likely in food animals as vancomycin resistant *E. faecalis* and *E. gallinarum/casseliflavus* were recovered from bovine clinical specimens [19]. As high-level vancomycin resistance of the VanA and VanB phenotypes is transferrable, and it has been reported in *E. gallinarum* and *E. casseliflavus* which are part of the normal flora in mammals [4], individuals in Uganda could be at risk of being colonized by such isolates via transmission from animals [4, 6]. Overall, our data reveals that speciation and accurate identification of VRE isolates in a resource-limited setting is important [4]. Moreover, because *E. gallinarum/casseliflavus* appear more frequent in human specimens [15, 16] and bovine specimens [19] in low-income countries compared to the developed countries [4–6], species-level identification of enterococci is key for instituting proper infection control measures and timely antibiotic therapy [6].

While the MDR frequency was low in this study, the isolates exhibited unique resistance patterns, for example ERY-GEN-AMP-CIP-MXF-TET (*E. faecalis*); ERY-AMP-NIT-MXF-TET (*E. durans*); VAN-TEC-LIZ-MXF (*E. gallinarum/casseliflavus*); VAN-TEC (*E. gallinarum/casseliflavus*). Thus, we have reported *E. gallinarum/casseliflavus* isolates that were resistant to both vancomycin and teicoplanin though they were susceptible to ampicillin. Antibiotic stewardship and strengthening of the infection control practices is necessary to prevent acquisition of ampicillin resistance in such strains. Lastly, cluster analysis of the Rep-PCR fingerprints revealed no evidence of clonal spread/transmission of enterococci in the Mulago Hospital setting though we used an inferior approach to infer genetic relatedness among isolates. However, this finding is in accordance with reports that VRE isolates in certain settings (e.g. the United States) are genetically diverse [4].

There were certain limitations in this study: First, a total of 47 stored enterococcal isolates mainly from vaginal swabs, urine and stool were not recovered on sub-culturing. However, as we succeeded in sub-culturing isolates from specimens where enterococci grew most (see Additional file 1: Table S1), we believe we avoided a potential selection bias. Second, we could not rule-out contamination: Given the high rate of colonization of health care workers by the enterococci, some of the isolates might have been not clinically relevant. Third, we did not investigate *E. gallinarum*/*casseliflavus* for *vanC* genes as these are chromosomally encoded hence inherent in these species. As well, our approach for isolate genetic relatedness was not robust and we recommend studies with superior approaches such as whole genome sequencing.

## Conclusions

Enterococci are frequent in clinical specimens at Mulago National Referral Hospital but they are susceptible to antibiotics commonly used to treat their infections e.g. ampicillin. As well, the frequency of MDR enterococci in the hospital is low and VRE isolates of *E. faecium* and *E. faecalis* are rare. However, non-*faecium* and non-*faecalis* VRE (*E. gallinarum*/*casseliflavus*) are frequent some with VanA and VanB high-level resistance to vancomycin. Thus, species-level identification of VRE isolates is important for instituting effective infection control measures that will curb the spread of VRE in the hospital.

## Additional files

Additional file 1: **Table S1.** Samples processed for isolation of bacteria. (DOCX 14 kb)

Additional file 2: **Figure S1.** Representative images (1% agarose gels) showing PCR detection of the *vanA* & *vanB* genes. *vanA*/*vanB* positive VRE isolates (*E. casseliflavus/gallinarum*) possessed the expected PCR product sizes i.e. 677 bp and 463 bp for *vanA* and *vanB*, respectively. Lanes in panel A depict: Lad, 100 bp ladder; Pos & Neg, vanA positive & negative controls, respectively; 1–7, samples of which 1, 2, 5, 6, 7 & 8 were vanA gene-positive while 3 & 4 were negative. Lanes in panel B depict: Lad, 100 bp ladder; Pos & Neg, *vanB* positive & negative controls, respectively; 1–7, samples of which 1, 2, & 3 were *vanB* gene-positive while 4 & 5 were negative (6 & 7 are repeats of 2 & 3). (TIFF 5313 kb)

## Data Availability

All data generated or analyzed during this study are included in this published article [and its additional files].

## Abbreviations

AMP: Ampicillin
AST: Antimicrobial susceptibility testing
BEA: Bile Esculin Agar
BHI: Brain heart infusion broth
CIP: Ciprofloxacin
DAP: Daptomycin
ERY: Erythromycin
GEN: Gentamicin
HGT: Horizontal gene transfer
ID: Identification
LIZ: Linezolid
MDR: Multidrug resistance
MIC: Minimum inhibitory concentration
MXF: Moxifloxacin
NIT: Nitrofurantoin
PCR: Polymerase chain reaction
SSI: Surgical site infection(s)
TEC: Teicoplanin
TET: Tetracycline
UTIs: Urinary tract infections
VAN: Vancomycin
VRE: Vancomycin resistant enterococci
