# Supplementary material for: Species, antibiotic susceptibility profiles and van gene frequencies among enterococci isolated from patients at Mulago National Referral Hospital in Kampala, Uganda
Source: BMC Infect Dis. 2019 May 31;19:486. doi: 10.1186/s12879-019-4136-7 (PMC6545014; doi:10.1186/s12879-019-4136-7)
Supplement: Supplementary file 1 — Table S1. Samples processed for isolation of bacteria. (DOCX 14 kb) [file 12879_2019_4136_MOESM1_ESM.docx]

**Table S1:**

Clinical samples (n=3229) processed for isolation of bacteria from 3229 patients at Mulago National Referral Hospital in Kampala, Uganda (2011-2012)

| **Specimen type** | **No. specimens (%)** | **No. specimens with enterococcus growth (%)^a^** | **Enterococcus recovery on sub-culturing (%)^b^** |
| --- | --- | --- | --- |
| Blood | 1094 (34) | 02 (0.18%) | 02 (100%) |
| Sputum | 777 (24) | 15 (1.9) | 15 (100) |
| High vaginal swabs | 287 (9) | 27 (9.4) | 0 (0) |
| Urine | 252 (7.8) | 32 (12.7) | 25 (78.1) |
| Endo-cervical swabs | 252 (7.8) | 02 (0.8) | 0 (0) |
| Stool | 238 (7.4) | 64 (27) | 61 (95.3) |
| Pus swabs | 133 (4.1) | 10 (7.5) | 10 (100) |
| Ear swabs | 42 (1.3) | 02 (4.8) | 02 (100) |
| Urethra swabs | 42 (1.3) | 07 (16.7) | 0 (0) |
| Cerebrospinal fluid | 42 (1.3) | 0 | - |
| Lymph node aspirate | 21 (0.65) | 0 | - |
| Oral swabs | 14 (0.43) | 0 | - |
| Rectal swabs | 07 (0.22) | 01 (14.3) | 0 (0) |
| Tracheal aspirate | 07 (0.22) | 0 | - |
| Broncho-alveolar lavage | 07 (0.22) | 0 | - |
| Pharyngeal swabs | 07 (0.22) | 0 | - |
| Pleural fluid | 07 (0.22) | 0 | - |
| **Total** | **3229** | **162 (5)** | **115 (71)** |

- **^a^**Primary processing of samples for detection of enterococci (2011-2012). The enterococcus were presumptively identified to genus level and pure isolates stored at -80C.
- **^b^**Recovery of stored enterococcus on sub-culturing (2015-2016): Isolates recovered represent a total of 115 specimens.
