# Supplementary figures and images for: Species, antibiotic susceptibility profiles and van gene frequencies among enterococci isolated from patients at Mulago National Referral Hospital in Kampala, Uganda
Source: BMC Infect Dis. 2019 May 31;19:486. doi: 10.1186/s12879-019-4136-7 (PMC6545014; doi:10.1186/s12879-019-4136-7)

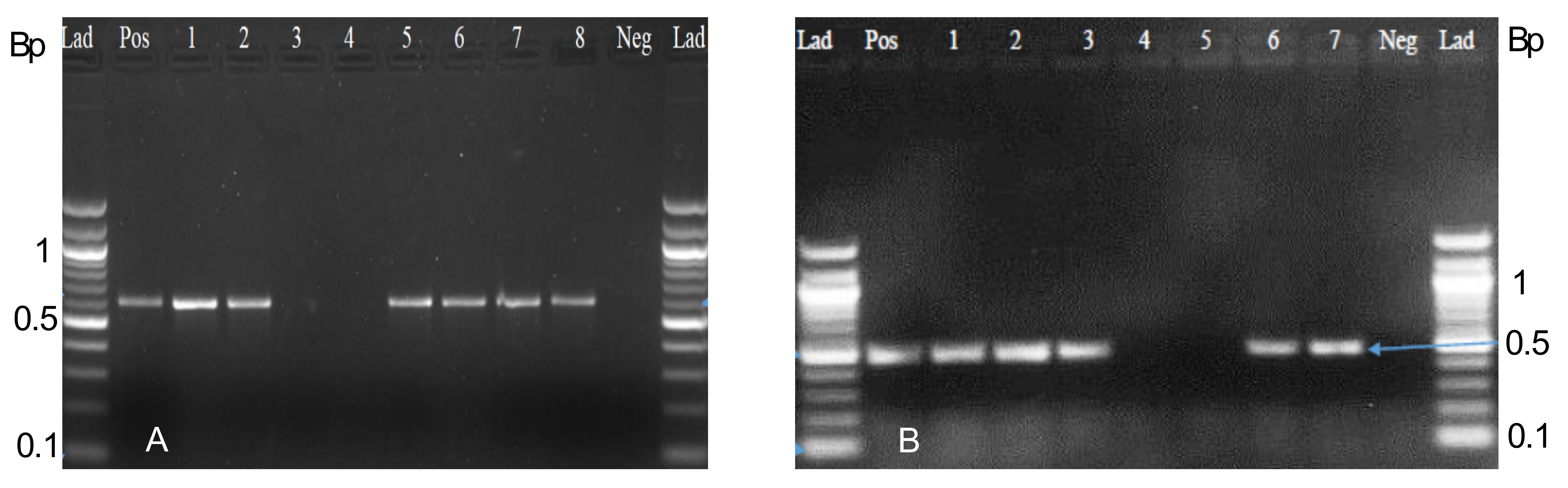

Supplement: Supplementary file 2 — Figure S1. Representative images (1% agarose gels) showing PCR detection of the vanA & vanB genes. vanA/vanB positive VRE isolates (E. casseliflavus/gallinarum) possessed the expected PCR product sizes i.e. 677 bp and 463 bp for vanA and vanB, respectively. Lanes in panel A depict: Lad, 100 bp ladder; Pos & Neg, vanA positive & negative controls, respectively; 1–7, samples of which 1, 2, 5, 6, 7 & 8 were vanA gene-positive while 3 & 4 were negative. Lanes in panel B depict: Lad, 100 bp ladder; Pos & Neg, vanB positive & negative controls, respectively; 1–7, samples of which 1, 2, & 3 were vanB gene-positive while 4 & 5 were negative (6 & 7 are repeats of 2 & 3). (TIFF 5313 kb) [file 12879_2019_4136_MOESM2_ESM.tiff]
